# Shared gene characteristics and molecular mechanisms of macrophages M1 polarization in calcified aortic valve disease

**DOI:** 10.3389/fcvm.2022.1058274

**Published:** 2023-01-04

**Authors:** Ming Qin, Qian Chen, Ning Li, Xiangyang Xu, Chuyi Wang, Guokun Wang, Zhiyun Xu

**Affiliations:** ^1^Department of Cardiovascular Surgery, Changhai Hospital, Naval Medical University, Shanghai, China; ^2^Department of Cardiothoracic Surgery, People’s Liberation Army Navy Medical Center, Naval Medical University, Shanghai, China

**Keywords:** calcific aortic valve disease (CAVD), M1 macrophages, bioinformatics, CCR7, hub genes

## Abstract

**Background:**

CAVD is a common cardiovascular disease, but currently there is no drug treatment. Therefore, it is urgent to find new and effective drug therapeutic targets. Recent evidence has shown that the infiltration of M1 macrophages increased in the calcified aortic valve tissues, but the mechanism has not been fully elucidated. The purpose of this study was to explore the shared gene characteristics and molecular mechanisms of macrophages M1 polarization in CAVD, in order to provide a theoretical basis for new drugs of CAVD.

**Methods:**

The mRNA datasets of CAVD and M1 polarization were downloaded from Gene Expression Omnibus (GEO) database. R language, String, and Cytoscape were used to analyze the functions and pathways of DEGs and feature genes. Immunohistochemical staining and Western Blot were performed to verify the selected hub genes.

**Results:**

CCR7 and GZMB were two genes appeared together in hub genes of M1-polarized and CAVD datasets that might be involved in the process of CAVD and macrophages M1 polarization. CCR7 and CD86 were significantly increased, while CD163 was significantly decreased in the calcified aortic valve tissues. The infiltration of M1 macrophages was increased, on the contrary, the infiltration of M2 macrophages was decreased in the calcified aortic valve tissues.

**Conclusion:**

This study reveals the shared gene characteristics and molecular mechanisms of CAVD and macrophages M1 polarization. The hub genes and pathways we found may provide new ideas for the mechanisms underlying the occurrence of M1 polarization during CAVD process.

## 1. Introduction

As the most prevalent valvular heart disease, calcific aortic valve disease (CAVD) is a major health problem with risk of severe morbidity and mortality (1). It ranges from valve thickening and mildly calcified to severe valve calcification with impaired leaflet motion and vast blood flow obstruction (2). In developed countries, calcific aortic stenosis is the second-most frequent cardiovascular disease after coronary artery disease and systemic arterial hypertension with a prevalence of 0.4% in the general population (3) and 1.7% in the population over 65 years (4). Currently, there is no effective medical treatment other than surgical or transcatheter aortic valve replacement (5, 6).

The aortic valve microenvironment mainly includes valvular interstitial cells, valvular endothelial cells, and immune cells (7). Recent evidence suggests that CAVD is an active process involving multiple complex pathological factors such as valvular endothelial cell injury, valvular interstitial cell differentiation, chronic inflammation, fibrosis, matrix remodeling, mechanical stress, and neovascularization (1, 8–11).

Currently, immune cells are also believed to play important roles in aortic valve calcification (12–14). Zhou’s study (15) showed that immune cells accounted for about 5.4% in the aortic valve tissue, and macrophages were the most dominant immune cells. Another study showed that (16) in the calcified aortic valves, the infiltration of M1 macrophages was increased, while the infiltration of M2 macrophages was decreased. Therefore, M1 macrophages may play an important role in the CAVD process (17).

At present, there are few studies on the mechanism of macrophages M1 polarization in CAVD. However, exploring the mechanism of macrophages M1 polarization in CAVD may help to prevent the occurrence of CAVD or delay the progression of CAVD. Microarray technology can effectively screen the biomarkers of CAVD. Gene Expression Omnibus (GEO)^1^ is a public database, and contains a large number of genes for various diseases. In this study, many bioinformatics analysis methods and microarray technology in GEO database were combined to screen and analyze the shared gene characteristics and molecular mechanism of macrophages M1 polarization in CAVD from the transcriptome level. This will be beneficial to deepen our understanding of CAVD and promote the research of therapeutic drugs for CAVD.

## 2. Materials and methods

### 2.1. Acquisition of GEO datasets

We use the keywords “calcified aortic valve disease” and “M1 macrophages” in GEO database (see text footnote 1) to search the mRNA datasets of CAVD and M1-polarized macrophages. The datasets we chose included mRNA microarray data of CAVD samples and healthy controls, as well as mRNA microarray data of M1 and M0 macrophages.

The GEO datasets were numbered as:

CAVD mRNA dataset: GSE51472, 10 CAVD samples and 5 control samples,
CAVD mRNA dataset: GSE83453 (10 samples of bicuspid aortic valve were excluded), 9 CAVD samples and 8 control samples,
M1-polarized mRNA dataset: GSE49240, 2 M1 macrophage samples and 2 M0 macrophage samples, culture condition of M0 macrophages: monocytes + M-CSF,
culture condition of M1 macrophages: M0 macrophages + IFN-γ + TNF-α,
M1-polarized mRNA dataset: GSE61298, 3 M1 macrophage samples and 3 M0 macrophage samples.
Culture condition of M0 macrophages: monocytes + M-CSF, culture condition of M1 macrophages: M0 macrophages + IFN-γ + LPS.

### 2.2. Microarray data processing

In this study, microarray datasets GSE49240 and GSE61298 were first used to identify differentially expressed genes (DEGs) during M1 polarization. The “limma” package in R language (version 4.1.3) was used to screen out DEGs in the process of M1 polarization. The cut-off values were |log2(foldchange)| > 1and *p*-value < 0.05. Then two other microarray datasets (GSE51472, GSE83453) were used to identify the feature modules in CAVD by WGCNA analysis. The overlapping DEGs between two M1 macrophage datasets and feature genes between two CAVD datasets were implemented by the “VennDiagram” package.

### 2.3. WGCNA analysis

Weighted gene co-expression network analysis (WGCNA) was an analytical method for analyzing gene expression patterns in multiple samples. It could cluster modules with similar expression and analyze the correlation between specific modules and disease phenotypes. In this paper, we used WGCNA analysis to obtain modules that were highly related to CAVD. We selected the top 5,000 genes according to their variance and analyzed them by the “WGCNA” package in R language. Before analysis, the “Hclust” function was first used for hierarchical cluster analysis to exclude the outlier samples. Then the “pickSoftThreshold” function in “WGCNA” package was used to select an appropriate power value (ranging from 1 to 30, with *R*^2^ ≥ 0.85) according to the criteria of the scale-free network. Then we constructed a hierarchical clustering dendrogram, clustered similar gene expressions into different modules, and calculated the correlation between each module and the disease phenotype. In this study, the soft threshold β of dataset GSE51472 was 19. The soft threshold β of dataset GSE83453 was 13. The other parameters were as followed: networkType = “unsigned,” minModuleSize = 30, mergeCutHeight = 0.25 and deepSplit = 2.

### 2.4. Functional enrichment analysis

Gene Ontology (GO) and Kyoto Encyclopedia of Genes and Genomes (KEGG) enrichment analysis were performed for Gene function and pathway analysis. GO enrichment analysis analyzed protein function at three levels: biological process, cellular component, and molecular function. GO and KEGG enrichment analysis were implemented by the “clusterProfiler” package. ClueGO enrichment analysis was a powerful complement to GO and KEGG enrichment analysis and was implemented by the ClueGO plug-in in Cytoscape (version: 3.7.1).

### 2.5. Protein-protein interaction network and hub genes screening

The construction of the protein-protein interaction (PPI) network was implemented by the online analysis tool String^2^ and Cytoscape. The cytoHubba plug-in in Cytoscape was used to evaluate the scores of gene nodes and screened out the top 20 hub genes (Top 20 genes were ranked by MCC method).

### 2.6. Cibersort immune infiltration analysis

Cibersort (cell-type identification by estimating relative subsets of RNA transcripts) was an immune infiltration algorithm based on transcriptome. It could calculate the relative content (LM22 gene signature was downloaded from https://www.nature.com/articles/nmeth.3337#MOESM207) and immune score (perm replacement = 1000, QN quantile normalization = TRUE) of 22 kinds of immune cells by using microarray or high-throughput sequencing data. On this basis, the Spearman correlation between hub genes and immune cells were calculated by the “ggcorrplot” package.

### 2.7. Analysis of single cell sequencing results

The single cell sequencing data in this paper was downloaded from Xu’s article (18)^3^ and included 9,410 CAVD cells and 3,366 normal aortic valve cells. The raw expression matrix was integrated into Seurat objects and filtered by the “Seurat” package. Then the “NormalizeData” function was used for normalization and the “UMAP” package was used for dimensionality reduction (FindClusters functions with a resolution of 0.5). Finally, cell clusters were annotated by the “singleR” package.

### 2.8. Clinical samples and histological examination

In this study, we collected aortic valve specimens from six patients who underwent aortic valve replacement or heart transplantation at the Department of Cardiovascular Surgery, Changhai Hospital Affiliated to Naval Medical University from April 2022 to July 2022. The study has been approved by the Ethics Review Committee of Changhai Hospital. Among the six patients, three patients who underwent aortic valve replacement were diagnosed with calcified aortic valve, and another three patients who underwent heart transplantation were diagnosed with dilated cardiomyopathy with normal aortic valve, which were identified by echocardiography and Alizarin red S staining. Patients with rheumatic heart disease, myocarditis, and bicuspid aortic valve were excluded. Part of the specimens were embedded in paraffin and the thickness of the paraffin section was 5 μm.

Alizarin red S staining: Paraffin sections were dewaxed and rehydrate routinely, Alizarin red S staining solution (1% dilution, pH = 4.2) was incubated at room temperature for 5 min, and then the sections were washed with anhydrous alcohol and sealed before observation.

Von Kassa (VK) staining: After dewaxing and hydration, paraffin sections were stained with silver nitrate, irradiated continuously with UV lamp for 1 h and washed with distilled water three times, and then sealed for observation.

Immunohistochemical (IHC) staining: The paraffin sections were dewaxed and hydrated before heat mediated antigen retrieval (Tris-EDTA buffer, pH = 9.0). Endogenous peroxidase activity was blocked by peroxidase blocking solution and non-specific binding sites were blocked by goat serum. Then the slides were incubated with the primary antibody overnight (4°C). The next day, biotin-labeled secondary antibody was added. Finally, DAB solution and hematoxylin were used for staining and counterstaining, respectively. Antibody Information: CCR7 (1:100 dilution, bioworld, USA, No. BS9847M), CD86 (1:100 dilution, Affibiotech, No. DF6332), CD163 (1:100 dilution, abcam, UK, No. ab182422).

### 2.9. Western blot analysis

Aortics valve specimens were lysed in SDS buffer containing a protease inhibitor PMSF (1:100 dilution) on ice for 30 min. Total protein concentrations were evaluated using a protein assay kit (Beyotime Biotech, Shanghai, China). Cell lysate was separated using 10% SDS-PAGE and then transferred to polyvinylidene difluoride membranes. Subsequently, the membranes were blocked for 1 h at room temperature by incubation in TBST solution containing 5% non-fat milk. Primary antibodies against CCR7 (1:1000 dilution, bioworld, USA, No. BS9847M), OPN (1:1000 dilution, Protein tech, Chicago, USA, No. 22952-1-AP), and GAPDH (1:50000 dilution, Protein tech, Chicago, USA, No.60004-1-Ig) were incubated overnight at 4°C. Finally, the membranes were incubated with appropriate secondary antibodies (Jackson Immuno Research, USA, 115-035-003, 1:5000) for 1 h at room temperature and detected with an ECL kit (Thermo Scientific, USA).

## 3. Results

### 3.1. Identification and analysis of DEGs in M1 macrophages datasets

#### 3.1.1. Identification of DEGs in M1 macrophages datasets

**FIGURE 1:**
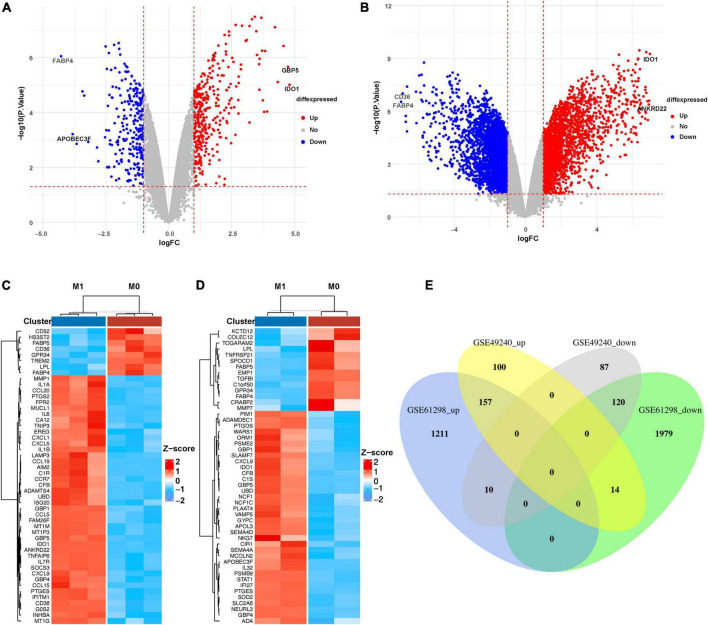
Identification of DEGs in M1 macrophages datasets. **(A)** Volcano diagram of DEGs in GSE49240 and **(B)** volcano diagram of DEGs in GSE61298, the cut-off value of –log10(*P*-value) is –log10(0.05), cut-off value of |log_2_fold change| is 1, red and blue, respectively, represent relatively high and low expression of the mRNA. **(C)** Heatmap of DEGs in GSE49240 and **(D)** heatmap of DEGs in GSE61298 showed the mRNA expression of M1 macrophages and M0 macrophages, with red representing up-regulation and blue representing down-regulation. The panels showed the top 50 DEGs ranked by |Log2fold change|. **(E)** Venn diagram of the overlapping DEGs from two datasets, GSE49240 and GSE61298, identified a total of 157 up-regulated and 120 down-regulated DEGs, with different colors representing different datasets.

M1-polarized mRNA datasets GSE49240 and GSE61298 were downloaded from GEO database. The “limma” package in R language was used to identify DEGs. 488 DEGs were screened from GSE49240 (including 271 up-regulated and 217 down-regulated mRNAs, shown in Supplementary Material 1). Three thousand four hundred and ninety-one DEGs were screened from the dataset GSE61298 (including 1378 up-regulated and 2113 down-regulated mRNAs, shown in Supplementary Material 1). The distribution of DEGs were visualized by volcano map in Figures 1A, B. At the same time, the top 50 DEGs ranked by |Log_2_fold change| were shown by heatmap in Figures 1C, D (mRNA expression levels were processed by Z-score). The results showed that IDO1 was the largest increased DEGs and FABP4 was the largest decreased DEGs in both datasets. In addition, the overlapping DEGs in the two M1-polarized datasets were visualized by Venn diagram (Figure 1E), and 277 overlapping DEGs were screened (including 157 up-regulated and 120 down-regulated mRNAs, shown in Supplementary Material 1).

#### 3.1.2. Enrichment analyses of the overlapping DEGs in M1-polarized datasets

**FIGURE 2:**
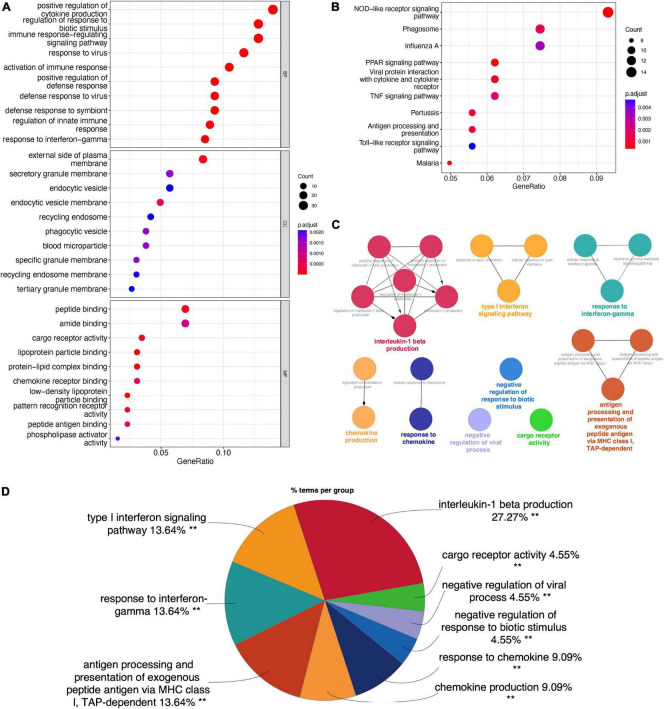
Enrichment analyses of the overlapping DEGs in M1-polarized datasets. **(A)** GO enrichment analysis of the overlapping DEGs, at three levels: biological process, cellular component, and molecular function. **(B)** KEGG pathway analysis of the overlapping DEGs. **(C)** The interaction network of GO terms, the significant term of each group was highlighted. **(D)** The proportion of each GO terms group in the total, **P < 0.05.

The overlapping DEGs were analyzed by GO, KEGG, and ClueGO enrichment analyses. GO enrichment analysis (Figure 2A) showed that DEGs were significantly associated with the biological processes of “Positive regulation of cytokine production,” “Regulation of response to biotic stimulus,” “Immune response-regulating signaling pathway,” “Response to virus,” and “Activation of Immune response.” KEGG pathway analysis (Figure 2B) showed that the overlapping DEGs were mainly enriched in “NOD-like receptor signaling pathway,” “Phagosome,” “Influenza A,” “PPAR Signaling Pathway,” and “Viral protein interaction with cytokine and cytokine receptor.” ClueGO enrichment analysis (Figure 2C) showed that the overlapping DEGs were mainly involved in “Interleukin-1 beta production,” “Type I interferon signaling pathway,” and “Response to interferon gamma.” The signaling pathway “Interleukin-1 beta production” accounted for 27.27% of the total terms, and 82 genes were involved (Figure 2D). These results suggested that chemokine-chemokine receptor pathway and inflammatory response may play important roles in macrophage M1 polarization.

#### 3.1.3. PPI analysis of the overlapping DEGs in M1-polarized datasets

**FIGURE 3:**
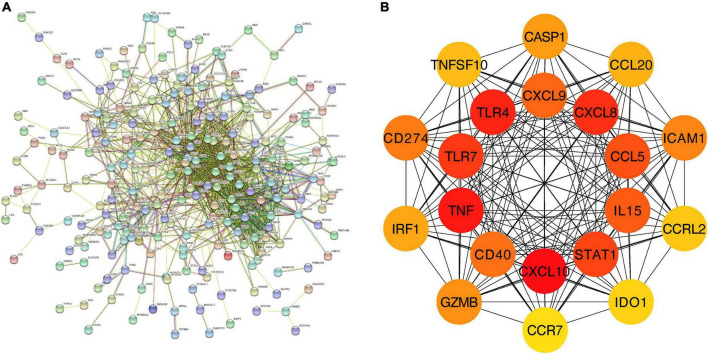
PPI network and hub genes of the overlapping DEGs. **(A)** The interaction between 277 overlapping DEGs. Minimum required interaction score = 0.4, 276 nodes and 1,107 edges were included in the PPI network. **(B)** Top 20 hub genes.

We further constructed a PPI network at the level of protein and screened out hub genes. Firstly, the online analysis tool String was used to analyze the interactions among 277 overlapping DEGs (minimum required interaction score = 0.4, 276 nodes and 1,107 edges were included in the PPI network) (Figure 3A). Then the cytoHubba plug-in in Cytoscape was used to calculate the score of gene nodes and screen out the top 20 hub genes. The results (shown in Supplementary Material 2) showed that CXCL10, TNF, TLR4, CXCL8, TLR7, STAT1, CCL5, IL15, CXCL9, CD40, CD274, ICAM1, GZMB, CASP1, IRF1, CCL20, TNFSF10, CCRL2, IDO1, and CCR7 were identified as hub genes (Figure 3B).

### 3.2. Identification and analysis of feature genes in CAVD datasets

#### 3.2.1. Identification of feature genes in CAVD datasets

**FIGURE 4:**
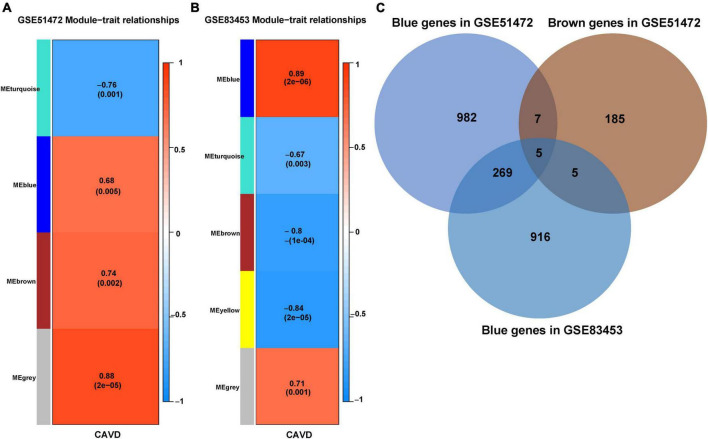
Identification of feature genes in CAVD datasets. **(A)** Module–phenotype relationships in CAVD dataset GSE51472. Each cell contains the corresponding correlation and *p*-value. **(B)** Module–phenotype relationships in CAVD dataset GSE83453. Each cell contains the corresponding correlation and *p*-value. **(C)** Venn diagram of the feature genes in two datasets, GSE51472 and GSE83453, identified a total of 279 up-regulated feature genes, with different colors representing different modules.

A total of five modules were identified by WGCNA analysis in CAVD mRNA dataset GSE51472. Then, a heatmap of module-phenotype relationship (Figure 4A) was drawn according to spearman correlation coefficient to evaluate the association between each module and disease. Two modules were selected as CAVD-related module (blue module: *r* = 0.68, *p* = 0.005, containing 1,263 genes; Brown module: *r* = 0.74, *p* = 0.002, containing 202 genes). Five modules were also identified in CAVD mRNA dataset GSE83453 (Figure 4B). Only the blue module was selected as CAVD-related module (blue module: *r* = 0.89, *p* = 2e^–06^, containing 1,195 genes).

CAVD-related modules in both datasets were shown in Supplementary Material 3. The CAVD-related modules in the two CAVD datasets were overlapped by Venn diagram. The results showed that there were 279 overlapping up-regulated genes between the two CAVD datasets, which may be associated with the pathogenesis of CAVD (Figure 4C, shown in Supplementary Material 3).

#### 3.2.2. Enrichment analyses of feature genes in CAVD datasets

**FIGURE 5:**
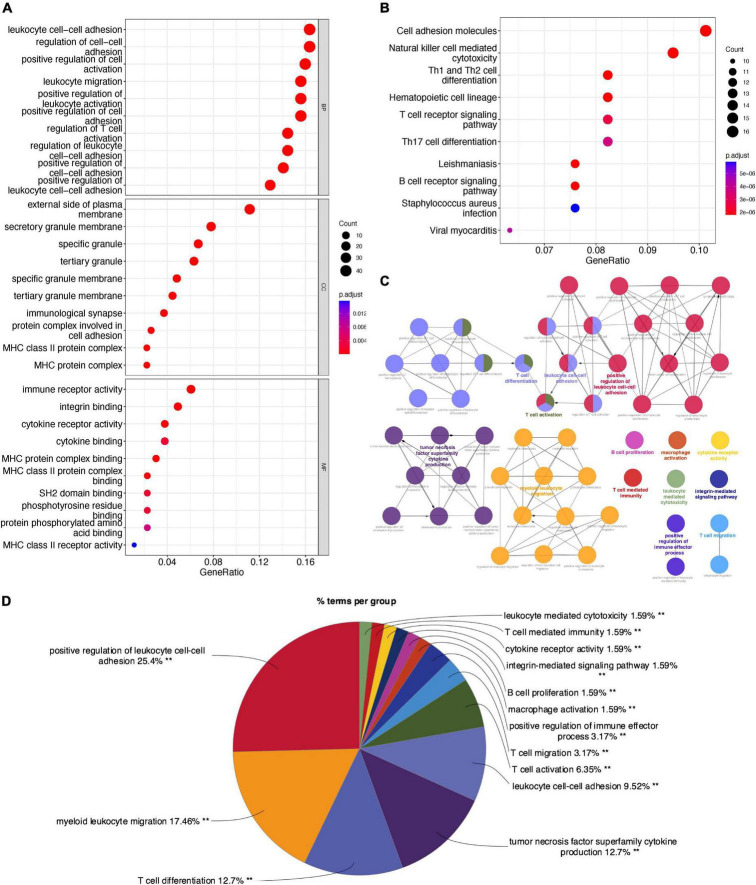
Enrichment analyses of feature genes in CAVD datasets. **(A)** GO enrichment analysis of the feature genes, at three levels: biological process, cellular component, and molecular function. **(B)** KEGG pathway analysis of the feature genes. **(C)** The interaction network of GO terms, the significant term of each group was highlighted. **(D)** The proportion of each GO terms group in the total, **P < 0.05.

The feature genes in two CAVD datasets were analyzed by GO, KEGG, and ClueGO enrichment analyses. GO enrichment analysis (Figure 5A) showed that the feature genes were significantly associated with the biological process of “Leukocyte cell-cell adhesion,” “Regulation of cell-cell adhesion,” “Positive regulation of cell activation,” “Leukocyte migration,” and “Positive regulation of leukocyte activation.” KEGG pathway analysis (Figure 5B) showed that the feature genes were mainly enriched in “Cell adhesion molecules,” “Natural killer cell mediated cytotoxicity,” “Th1 and Th2 cell differentiation,” “Hematopoietic cell lineage,” and “T cell receptor signaling pathway.” ClueGO enrichment analysis (Figure 5C) showed that the feature genes were mainly involved in “Positive regulation of leukocyte cell-cell adhesion,” “Myeloid leukocyte migration,” and “T cell differentiation.” The signaling pathway “Positive regulation of leukocyte cell-cell adhesion” accounted for 25.4% of the total terms, and 479 genes were involved (Figure 5D). These results suggested that the cell-cell adhesion pathway may play an important role in CAVD.

#### 3.2.3. PPI analysis of feature genes in CAVD datasets

**FIGURE 6:**
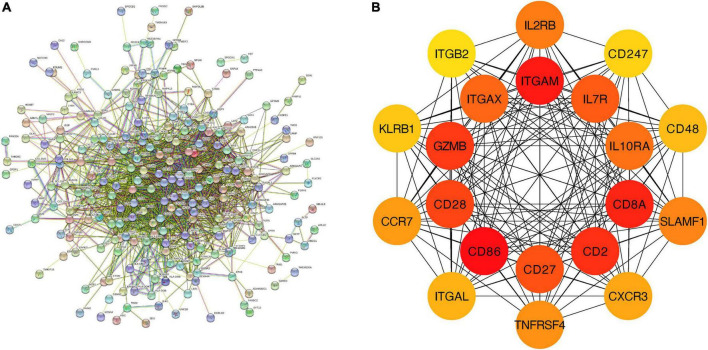
PPI network and hub genes of feature genes. **(A)** The interaction between 279 feature genes. Minimum required interaction score = 0.4, 275 nodes and 1,705 edges were included in the PPI network. **(B)** Top 20 hub genes.

We further constructed a PPI network at the level of protein and screened out hub genes. Firstly, String was used to analyze the interactions among 279 feature genes (minimum required interaction score = 0.4, 275 nodes and 1,705 edges were included in the PPI network) (Figure 6A). Then the cytoHubba plug-in was used to calculate the score of gene nodes and screen out the top 20 hub genes. The results (shown in Supplementary Material 4) showed that CD86, ITGAM, CD8A, CD2, GZMB, CD28, CD27, IL7R, ITGAX, IL10RA, IL2RB, SLAMF1, TNFRSF4, CCR7, CXCR3, ITGAL, CD48, KLRB1, CD247, and ITGB2 were identified as hub genes (Figure 6B).

### 3.3. Identification and analysis of shared genes between M1-polarized and CAVD datasets

#### 3.3.1. Identification of shared genes between M1-polarized and CAVD datasets

**FIGURE 7:**
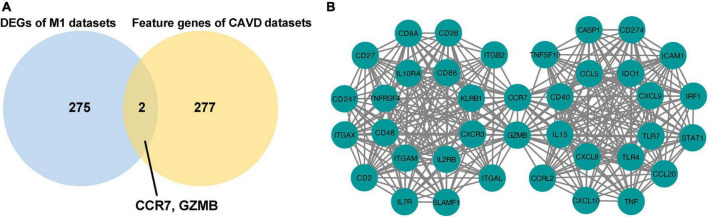
Identification of shared genes between M1-polarized and CAVD datasets. **(A)** Venn diagram of the shared genes in M1-polarized and CAVD datasets. **(B)** The interaction between 40 hub genes in M1-polarized and CAVD datasets.

The overlapping DEGs in M1-polarized datasets were overlapped with the feature genes in CAVD datasets. The results showed that CCR7 and GZMB appeared simultaneously (Figure 7A), and both were hub genes in the CAVD datasets and the M1-polarized datasets (Figure 7B). These results suggested that CCR7 and GZMB may play important roles in M1 polarization and CAVD.

#### 3.3.2. Immune infiltration analysis of CAVD datasets

Next, two CAVD datasets were analyzed for immune infiltration. The results showed that compared with the normal aortic valves, the proportion of M0 and M1 macrophages were increased, but the proportion of M2 macrophages was decreased in the calcified aortic valves (Supplementary Figures 1, 2). By calculating the correlation between hub genes and immune cells in CAVD datasets, this study found that CCR7 was positively correlated with M1 macrophages, while GZMB was negatively correlated with M1 macrophages (Supplementary Figures 3, 4). Therefore, we focused on the relationship between CCR7 and M1 macrophages.

#### 3.3.3. Distribution of CCR7 in aortic valve tissue cells

**FIGURE 8:**
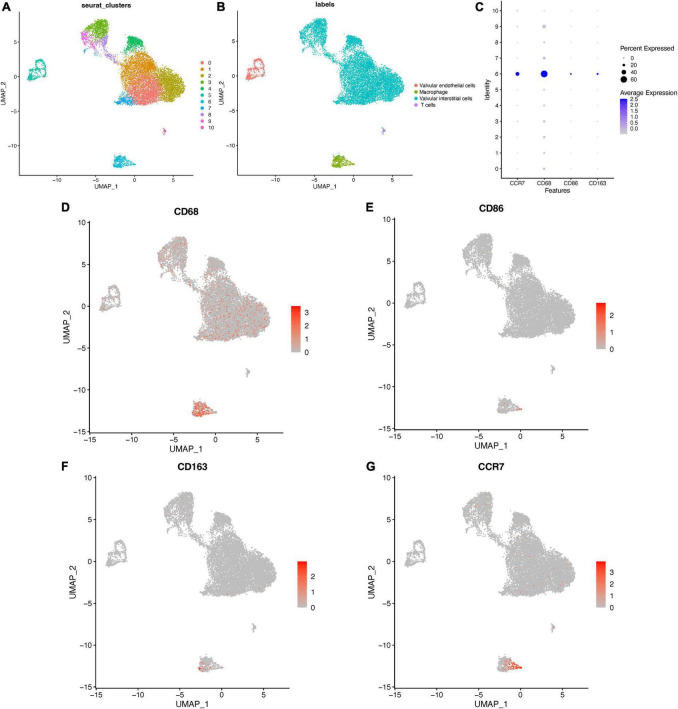
Distribution of CCR7 in aortic valve cells. **(A)** The clusters of cells in aortic valve tissues and the annotation of cells **(B)** in aortic valve tissues. **(C)** The bubble map of the expressions of different markers in aortic valve cells. **(D–G)** The distribution of different markers in aortic valve cells.

To investigate cellular heterogeneity in aortic valves microenvironment at single-cell resolution, aortic valve tissue cells were divided into 11 clusters (Figure 8A, FindClusters functions with a resolution of 0.5) by using the “UMAP” package. The annotation of single cell sequencing (Figure 8B) showed that the aortic valve tissue included four types of cells, including valvular interstitial cells (VICs), valvular endothelial cells, T cells, and macrophages. Among them, most of the cells in Cluster 6 were macrophages. The results of bubble map (Figure 8C) showed that CCR7, CD68 (macrophage marker), CD86 (M1 macrophage marker), and CD163 (M2 macrophage marker) were significantly higher in Cluster 6 than those in other clusters. Then the distribution of M1 and M2 macrophages were marked by CD86 and CD163, respectively. The results showed that the distribution of CCR7 was consistent with that of CD86, but not consistent with that of CD163 (Figures 8D–G). These results indicated that CCR7 was associated with M1 macrophages.

### 3.4. Experimental verification of clinical specimens

#### 3.4.1. Pathological verification of calcification of clinical specimens

**FIGURE 9:**
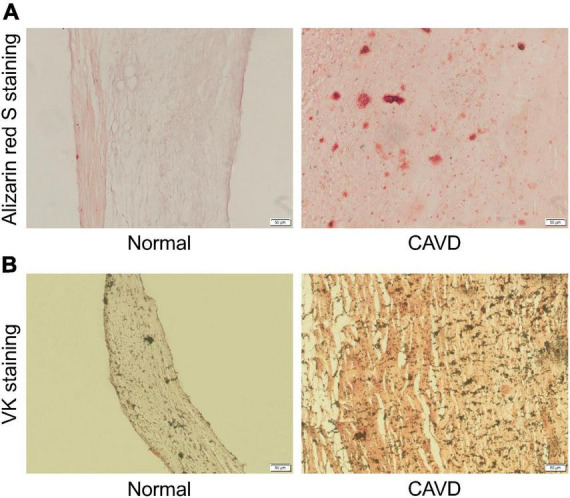
Representative Alizarin red S staining and VK staining images. **(A)** Representative Alizarin red S staining images of normal aortic valve tissue and calcified aortic valve tissue. **(B)** Representative VK staining images of normal aortic valve tissue and calcified aortic valve tissue.

Aortic valves from patients with calcified or normal aortic valves were selected and sectioned. Calcified nodules were identified by Alizarin red S staining and VK staining in calcified aortic valve samples. Representative Alizarin red S staining and VK staining images were shown as follows (Figures 9A, B).

#### 3.4.2. IHC staining and western blot verification of clinical specimens

**FIGURE 10:**
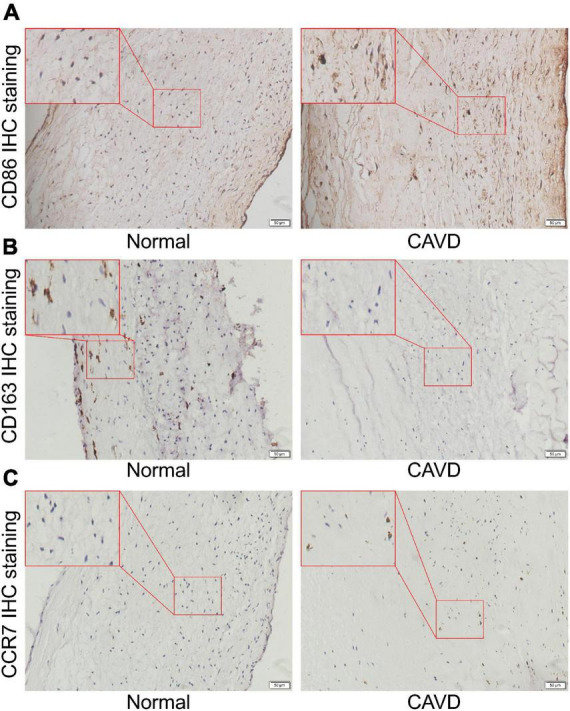
Representative IHC staining images. **(A)** Representative CD86 IHC staining images of normal aortic valve tissue and calcified aortic valve tissue. **(B)** Representative CD163 IHC staining images of normal aortic valve tissue and calcified aortic valve tissue. **(C)** Representative CCR7 IHC staining images of normal aortic valve tissue and calcified aortic valve tissue.

Finally, the calcified and normal aortic valve tissues were sectioned for CCR7, CD86, and CD163 IHC staining. The IHC staining results showed the expression level of CCR7 and CD86 were significantly increased in the calcified aortic valve tissues compared to the normal aortic valve. On contrary, the expression of CD163 was significantly decreased as shown in Figures 10A–C. We further investigated the expression profile of CCR7 in calcified and normal aortic valves tissues. Western blot assay confirmed that the expression level of CCR7 was increased in calcified aortic valve tissues (Supplementary Figure 5).

## 4. Discussion

In this study, we screened out the DEGs in M1 polarization. Enrichment analyses showed that chemokine-chemokine receptor pathway and inflammatory response may play important roles in the process of M1 polarization. Then, the feature genes of CAVD were screened out. Enrichment analyses indicated that cell-cell adhesion pathway may play an important role in the process of CAVD. CCR7 and GZMB appeared in hub genes of M1-polarized and CAVD datasets. These results suggested that CCR7 and GZMB may regulate CAVD by regulating the polarization, chemotaxis, and adhesion of M1 macrophages. Cibersort immune infiltration analysis showed that the infiltration of M1 macrophages was increased and the infiltration of M2 macrophages was decreased during the CAVD process. Single cell sequencing results showed that the distribution of CCR7 was consistent with the distribution of CD86, but not with CD163, suggesting that CCR7 was correlated with M1 macrophages, which may affect the process of CAVD. IHC staining results showed that, compared with the normal aortic valve tissues, the expressions of CCR7 and CD86 were significantly increased in the calcified aortic valve tissues, while the expression of CD163 was significantly decreased. These results indicated that the infiltration of M1 macrophages was increased and the infiltration of M2 macrophages was decreased. Western blot assay further confirmed that CCR7 was increased in calcified aortic valve tissues.

Recent studies have shown that macrophage play an important role in CAVD (19), the infiltration of M1 macrophages was increased and the infiltration of M2 macrophages was decreased in the calcified aortic valves (20). Raddatz et al.’s (21) research reported that increased macrophage recruitment promotes osteogenic calcification. Compared with unstimulated conditioned macrophage medium, conditioned medium of M1 macrophage enhanced expression of osteogenic genes in VICs, while inhibition of M1 polarization inhibited osteoblastic differentiation in VICs, suggesting that M1 macrophage may promote human aortic valve calcification (17, 22, 23). Grim et al.’s article reported that proinflammatory cytokines in M1 conditioned media inhibit myofibroblast activation in VICs and promote their osteogenic differentiation (24).

The protein encoded by CCR7 is a member of the G protein-coupled receptor family (25–27) and is up-regulated in response to inflammation, pathogens, and tissue damage (28). CCR7 chemokine axis is composed of chemokine ligands CCL19 and CCL21, and chemokine receptor CCR7 (29, 30). At the cellular level, CCR7-mediated signaling pathways mainly include the activation of integrins leading to cell adhesion and the polarization of actin cytoskeleton (31). Activation of CCR7 can activate T cells, secrete a large amount of IFN-γ, and promote Th1 polarization (32). The deficiency of CCR7 leads to Th2 polarization and activation of B cells (33). CCL19 and CCL21 only induce M1 polarization, and CCL19 or CCL21 induced activation of both MEK1-ERK1/2 and PI3K-AKT cascades in M1 but not in M2 macrophages (27). In rheumatoid arthritis, it has been reported that the activation of CCL19/CCL21-CCR7 signaling pathway increases the infiltration of M1 macrophages (34, 35). It has been shown that patients with symptomatic aortic stenosis have higher serum CCL21 level compared to healthy controls (36). Another study reported that O_2_ consumption and CO_2_ production were higher in CCR7^–/–^ mice than in wild-type mice. CCR7^–/–^ mice are protected from diet-induced obesity and subsequent insulin resistance (37), which may protect CCR7^–/–^ mice from CAVD. This study hypothesized that the CCR7 axis may promote the polarization and adhesion of macrophages, thereby promoting the progression of CAVD.

GZMB encodes a member of the granzyme subfamily of proteins, granzyme B. GZMB-induced apoptotic cells, when phagocytosed by macrophages, can induce the production of TGF-β and thereby influence the Th1/Th2 cytokine and Ig balance (38). Another research reported that GZMB mediates IL-8/macrophage inflammatory protein-2 secretion (39). Further, Guo et al.’s study also reported that GZMB was differentially expressed gene in CAVD datasets (40), which supports our analysis.

To sum up, CCR7 may act as a potential target for the treatment of CAVD. This study still has many limitations. At first, the increased expressions of CCR7 and M1 macrophages in calcified aortic valve tissues were verified only by bioinformatics analyses and clinical specimens in this study. The function of CCR7 needs to be explored by a more comprehensive design, both *in vitro* and *in vivo*, to determine its in-depth mechanism leading to CAVD. Secondly, the sample size of the datasets in this paper are too small, which may reduce the reliability of the analysis results. Analysis of datasets with larger sample size and more detailed description of patient characteristics will facilitate the understanding of the results. At last, our grouping of the samples in the dataset GSE51472 is not optimal. Putting mildly calcified and severely calcified samples together may result in false negative results. We will pay more attention to the quality of our datasets and achieve homogeneity in the same group of samples in the future.

## 5. Conclusion

In this study, bioinformatics analyses suggested that CCR7 and GZMB might be involved in the process of CAVD. In addition, the infiltration of M1 macrophages was increased and the infiltration of M2 macrophages was decreased during the CAVD process, and the distribution of CCR7 was consistent with that of M1 macrophages. These results suggest that CCR7 may regulate the process of CAVD by regulating macrophage polarization and adhesion.

## Data availability statement

The datasets presented in this study can be found in online repositories. The names of the repository/repositories and accession number(s) can be found in the article/Supplementary material.

## Ethics statement

The studies involving human participants were reviewed and approved by the Ethics Review Committee of Changhai Hospital. The patients/participants provided their written informed consent to participate in this study. Written informed consent was obtained from the individual(s) for the publication of any potentially identifiable images or data included in this article.

## Author contributions

ZX and GW designed the study. MQ and NL analyzed the data and finished the manuscript. QC, CW, and XX completed the experiments. All authors contributed to the article and approved the submitted version.

## Footnotes

1
https://www.ncbi.nlm.nih.gov/geo/

2
https://cn.string-db.org

3
https://www.jianguoyun.com/p/DRQVbtkQp8_2CRiVrZME
