# Supplementary material for: Shared gene characteristics and molecular mechanisms of macrophages M1 polarization in calcified aortic valve disease
Source: Front Cardiovasc Med. 2023 Jan 4;9:1058274. doi: 10.3389/fcvm.2022.1058274 (PMC9846331; doi:10.3389/fcvm.2022.1058274)
Supplement: Supplementary file 1 [file Data_Sheet_1.ZIP › Qin Supplementary material/Supplementary_Figure.docx]

Supplementary Material

# Supplementary Figures

**Supplementary Figure 1.** Box plots of immune infiltration in CAVD dataset GSE51472.

**Supplementary Figure 2.** Box plots of immune infiltration in CAVD dataset GSE83453.


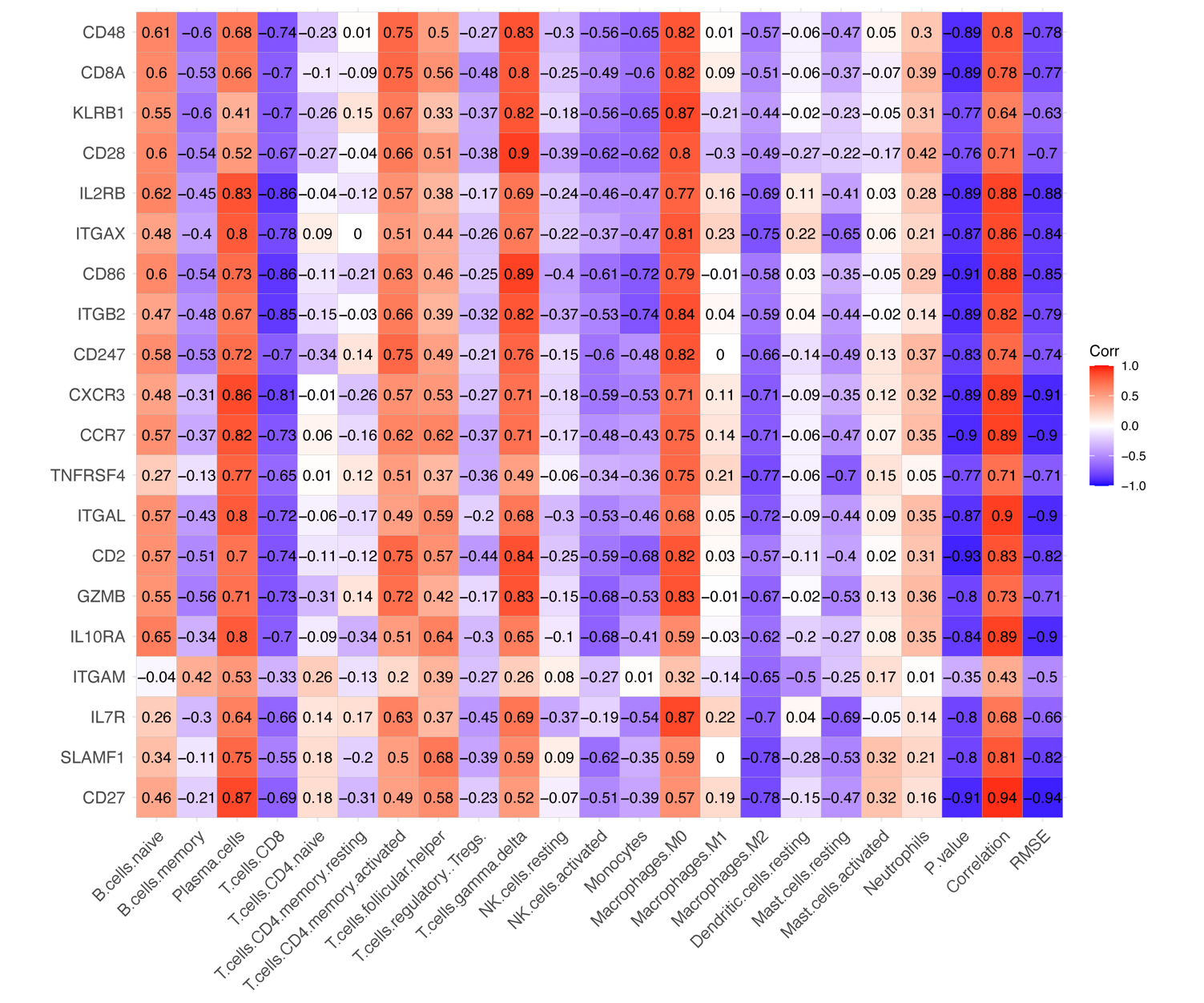


**Supplementary Figure 3.** The correlation between hub genes and immune cells in CAVD dataset GSE51472. Red represents positive correlation and blue represents negative correlation.


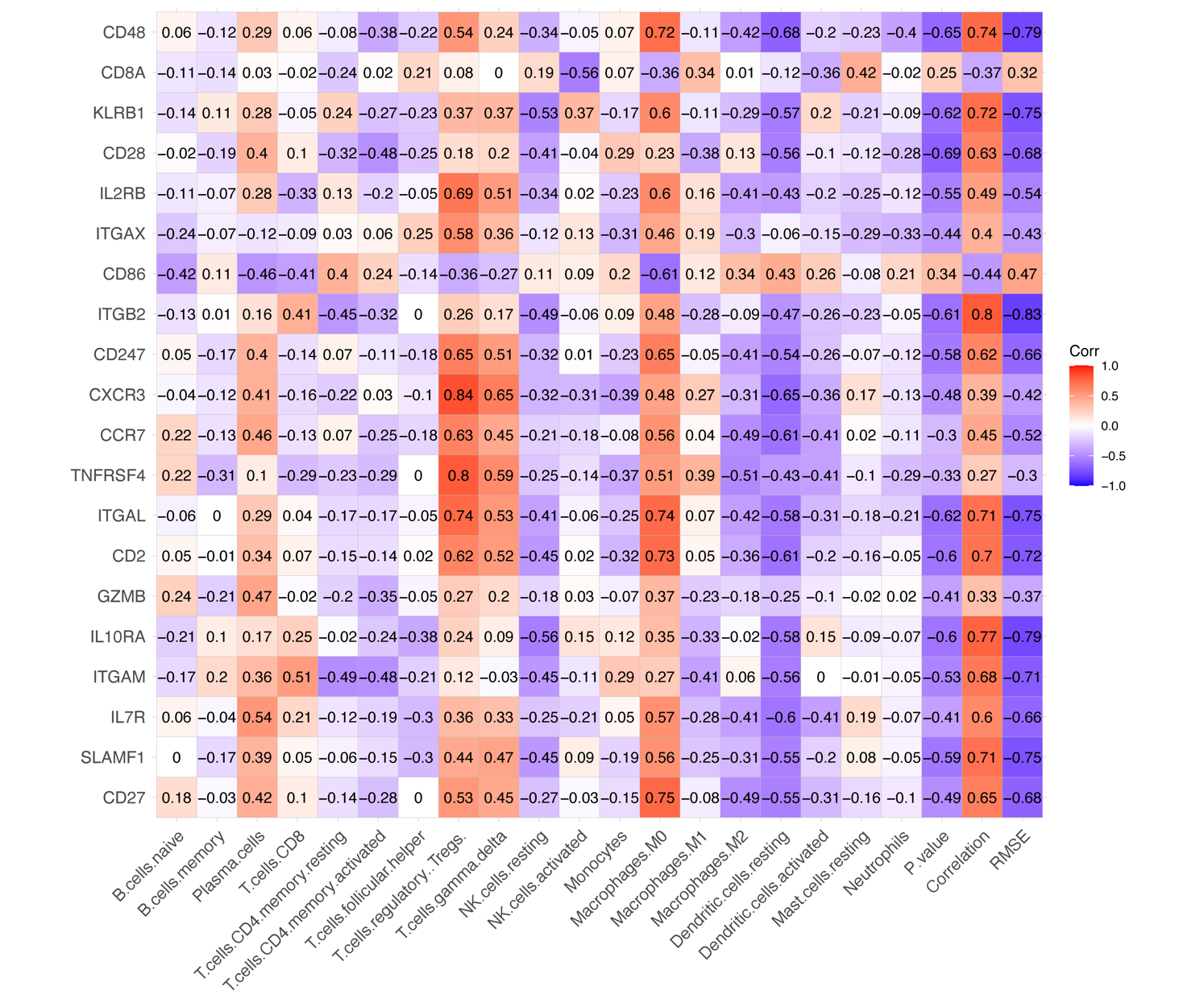


**Supplementary Figure 4.** The correlation between hub genes and immune cells in CAVD dataset GSE83453. Red represents positive correlation and blue represents negative correlation.


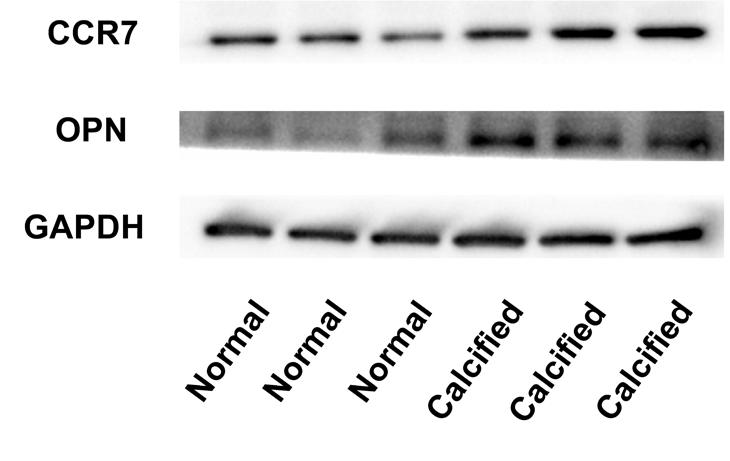


**Supplementary Figure 5. CCR7 expression profile in normal and calcified aortic valve tissues**
